# Genetic Characterization and Phylogenetic Analysis of Fasciola Species Isolated From Yaks on Qinghai-Tibet Plateau, China

**DOI:** 10.3389/fvets.2022.824785

**Published:** 2022-05-12

**Authors:** Xing Gao, Dongjing Wang, Zhao Zhang, Chuxian Quan, Shimeng Zhou, Kewei Li, Yan Li, Suonan Zhao, Xiangying Kong, Muhammad Fakhar-e-Alam Kulyar, Jiangyong Zeng, Jiakui Li

**Affiliations:** ^1^College of Veterinary Medicine, Huazhong Agricultural University, Wuhan, China; ^2^Tibet Livestock Research Institute, Tibet Academy of Agricultural and Animal Husbandry Sciences, Lhasa, China; ^3^State Key Laboratory of Hulless Barley and Yak Germplasm Resources and Genetic Improvement, Lhasa, China; ^4^Haibei Agricultural and Animal Husbandry Sciences Institute, Haibei, China; ^5^College of Animals Husbandry and Veterinary Medicine, Tibet Agricultural and Animal Husbandry University, Linzhi, China

**Keywords:** yaks, *Fasciola intermediate*, mitogenome, gene order, phylogenetic

## Abstract

The present study determined the complete mitochondrial DNA (mt DNA) sequence of *Fasciola intermediate* (isolated from yaks) based on gene content and genome organization. According to our findings, the genome of *Fasciola intermediate* was 13,960 bp in length, containing 2 ribosomal RNA (rRNA) genes, 12 protein-coding genes (PCGs), and 22 transfer RNA (tRNA) genes. The A+T content of genomes was 63.19%, with A (15.17%), C (9.31%), G (27.51%), and T as the nucleotide composition (48.02%). Meanwhile, the results showed negative AT-skew (-0.52) and positive GC-skew (0.494). The AT bias significantly affected both the codon usage pattern and amino acid composition of proteins. There were 2715 codons in all 12 protein-coding genes, excluding termination codons. Leu (16.72%) was the most often used amino acid, followed by Val (12.74%), Phe (10.90%), Ser (10.09%), and Gly (8.39%). A phylogenetic tree was built using Maximum-Likelihood (ML) through MEGA 11.0 software. The entire mt DNA sequence of *Fasciola intermediate* gave more genetic markers for investigating Trematoda population genetics, systematics, and phylogeography. Hence, for the first time, our study confirmed that yaks on the Qinghai-Tibet plateau have the infestation of *Fasciola intermediate* parasite.

## Introduction

Yak is a unique bovine specie on the Qinghai-Tibet plateau, China. The Qinghai-Tibet plateau is home to around 14 million yaks (a small amount of distribution in India, Bhutan, Sikkim, Afghanistan and Pakistan) (1). The yaks are necessary for herders in this area because of their milk, wool, and meat (2). Fasciolosis is one of the most important parasitic zoonotic issue, mainly caused by *Fasciola hepatica* (*F. hepatica*)*, Fasciola gigantica* (*F. gigantica*) (3, 4). The infection occurs mostly through the oral route (5), causing emaciation, fever, hepatomegaly, cholangitis, and jaundice, and even death (5). Approximately 2.4 million humans and more than 600 million animals are infected per year, causing serious public health threats and considerable economic loss to the livestock industry (6, 7).

The sequence of mitochondrial DNA (mt DNA) is an extrachromosomal genome, commonly used as an informative genetic marker for various evolutionary studies among species due to the maternal inheritance. It includes molecular evolution, comparative population genetics, phylogenetics, and evolutionary genomics (8, 9). In most parasites, mitogenomes are ~13 kilobases (Kb) in size as closed circular molecules (10). The mt DNA contains 36 genes, including 12 protein coding genes (PCGs) (subunits 6 of the ATPase (*atp6*), cytochrome B (*cob*), cytochrome c oxidase subunits 1–3 (*cox1*–*cox3*), NADH dehydrogenase subunits 1–6 and 4 L (*nad1–6* and *nad4L*), two ribosomal RNA genes encoding the small and large subunit rRNAs (*rrnL* and *rrnS*), 22 transfer RNA (tRNA) genes and a control region (CR) of variable length, known as the A+T-rich region (11, 12).

Previous research has identified the species of *Fasciola* in buffaloes and sheep, but not in yaks of the Qinghai-Tibet plateau. Therefore, in this study, the complete mitogenome of *F. intermediate* from yaks was initially sequenced and compared with other Trematoda mitogenomes (4). The available complete mitogenomes were used to provide insight into the phylogenetic relationship of *F. intermediate*. These findings would be valuable in order to have a better understanding of the *F. intermediate* mitogenome and the evolutionary relationships within Trematoda. The characteristics of *F. intermediate* mitogenome might be used to build appropriate genetic markers to detect Fasciola in yaks. This study might provide a basis for the accurate prevention, diagnosis, and treatment of Fasciolosis in yaks for studying the population genetic structure of *F. intermediate* in China.

## Materials and Methods

### Samples and DNA Extraction

The *F. intermediate* samples used in this study were collected from the chopped liver of yaks (Xiahua slaughter house in Tibetan Autonomous Prefecture of Haibe, Qinghai Province, China). The samples were fixed in 75% alcohol and stored at −20°C until used for DNA extraction. Total genomic DNA was isolated by using TIANcombi DNA Lyse&Det PCR Kit [TIANGEN biotech (Beijing) Co., Ltd.].

### Gene Annotation and Sequence Analysis

DNA samples were randomly interrupted, the required length of DNA fragments were collected, the base “A” to 3′-end was added, DNA fragments and 3′-end were connected with “T” base special joint, and finally used for cluster preparation and sequencing.

The mitogenome of *F. intermediate* was sequenced by next-generation sequencing (NGS). Two lanes for *F. intermediate* were sequenced as 400 bp reads using Illumina MiSeq (1 GB raw). The raw data was saved by Paired-End FASTQ and generated high-quality sequences by using AdapterRemoval (version 2) and SOAPec (version 2.01) based on K-mer distribution (13).

A5-miseqv20150522 and SPAdesv3.9.0 were used to assemble high-quality second-generation sequencing data to construct contig and scaffold sequences (14, 15). Mitochondrial sequences of each splicing result were selected by Blastn (BLAST v2.2.31+), compared between the sequences with high sequencing depth and the NT library on NCBI. By mummerv3.1 software. The results of mitochondrial splicing were used for collinearity analysis. The location relationship was determined, and then gap between contig was filled (16).

The complete mitochondrial genome sequences of splicing were uploaded to MITOS web server (http://mitos.bioinf.uni-leipzig.de/) for functional annotation (17). The Genetic Code was set to 05-invertebrate; the rest was set as the default parameter by MITOS. The secondary structure of each predicted tRNA can be obtained in the MITOS web server. The complete genome circle map of mitochondria was drawn using CGview visualization software (18).

### Phylogenetic Analysis

The taxonomic status of *F. intermediate* with available Trematoda was estimated by reconstructing phylogenetic trees. The complete nucleotide sequences of 36 Trematoda and one outgroup are available at GenBank (https://www.ncbi.nlm.nih.gov/genbank/).

Nucleotide sequences of each gene and their deduced amino acid sequences were aligned separately by using https://www.novoprolabs.com/ and MEGA 11.0 (19). The phylogenetic tree was built using Maximum-Likelihood (ML) through MEGA 11.0 software. The genome information used in this study is shown in Table 1.

**Table 1:** Position and nucleotide sequence lengths of mitochondrial genomes of *F. intermediate*, and start and stop codons for protein-coding genes as well as their tRNA gene anticodons.

**Feature**	**Strand**	**Position**	**Length (bp)**	**Initiation codon**	**Stop codon**	**Anticodon**	**Intergenic nucleotide**
cox3	N	231–633	403	ATG	TAA	-	15
trnH	N	649–713	65	-	-	GTG	0
cob	N	714–1770	1,057	ATA	T	-	64
nad4l	N	1,835–2,099	265	ATG	TAA	-	133
nad4	N	2,233–3,172	940	TAG	TAA	-	165
trnQ	N	3,338–3,404	67	-	-	TTG	11
trnF	N	3,416–3,482	67	-	-	GAA	4
trnM	N	3,487–3,553	67	-	-	CAT	47
atp6	N	3,601–4,054	454	ATC	TAA	-	62
nad2	N	4,117–4,624	508	ATT	TAA	-	331
trnV	N	4,956–5,020	65	-	-	TAC	13
trnA	N	5,034–5,099	66	-	-	TGC	2
trnD	N	5,102–5,167	66	-	-	GTC	65
nad1	N	5,233–6028	796	ATT	TAG	-	54
trnN	N	6,083–6,153	71	-	-	GTT	8
trnP	N	6,162–6,230	69	-	-	TGG	−2
trnI	N	6,229–6,293	65	-	-	GAT	2
trnK	N	6,296–6,363	68	-	-	CTT	−1
nad3	N	6,363–6,711	349	ATA	TAG	-	12
trnS1	N	6,724–6,780	57	-	-	GCT	8
trnW	N	6,789–6,852	64	-	-	TCA	2
cox1	N	6,855–8,310	1,456	ATG	TAA	-	98
trnT	N	8,409–8,477	69	-	-	TGT	441
rrnL	N	8,919–9,446	528	-	-	-	15
trnC	N	9,462–9,532	71	-	-	GCA	−3
rrnS	N	9,530–10,274	745	-	-	-	47
cox2	N	10,322–10,910	589	ATG	TAA	-	36
nad6	N	10,947–11,376	430	ATA	TAA	-	30
trnY	N	11,407–11,474	68	-	-	GTA	−1
trnL1	N	11,474–11,539	66	-	-	TAG	−3
trnS2	N	11,537–11,600	64	-	-	TGA	5
trnL2	N	11,606–11,670	65	-	-	TAA	4
trnR	N	11,675–11,733	59	-	-	TCG	208
nad5	N	11,942–12,854	913	ATT	TAA	-	468
trnE	N	13,323–13,391	69	-	-	TTC	175
trnG	N	13,567–13,631	65	-	-	TCC	-

## Result and Discussion

### Genome Organization and Nucleotide Composition

**Figure 1:**
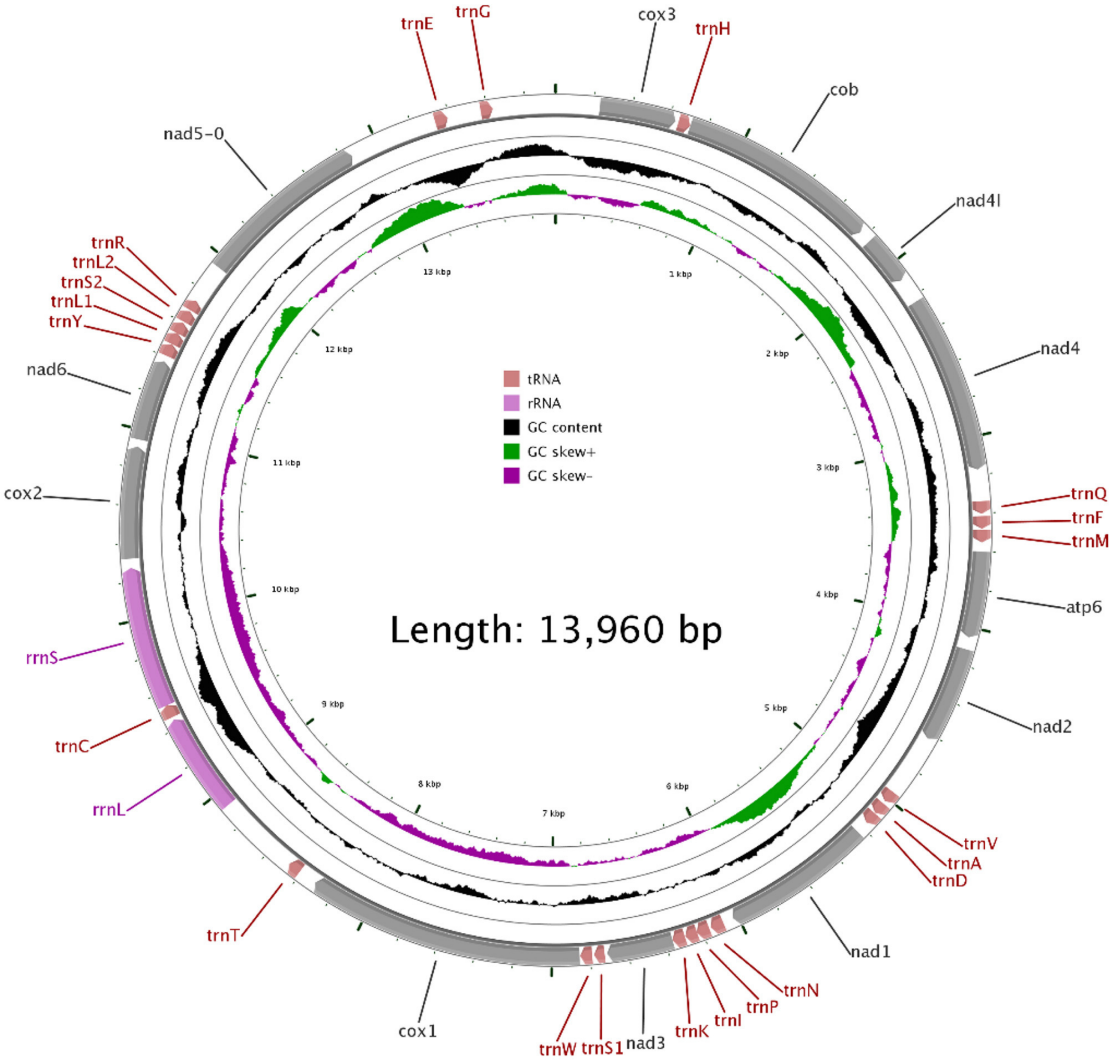
The mitochondrial genome of *F. intermediate* from yak.

In our study, the mitogenome of *F. intermediate* was a closed circular molecule of 13,960 bp in length (Figure 1). The mitogenome contained 36 typical mitochondrial genes [12 PCGs (cox1-3, nad1-6, nad4L, cob and atp6), 22 tRNAs, 2 rRNAs (rrnS and rrnL)] (Table 2). The mitogenome of *F. intermediate* had been submitted to the NCBI GenBank under the accession number MH621335.1. The nucleotide compositions of the mitogenome of *F. intermediate* were as follows: A = 15.17%; T = 48.02%; G = 27.51%; and C = 9.31%. The whole mitogenome of *F. intermediate* was biased toward AT nucleotides (63.19%) (Table 3). All genes were encoded on the minority (N) strand (Table 2). There was a 10 bp overlap between genes in five locations: trnP/trnI, trnK/nad3, trnC/rrnS, trnY/trnL1, and trnL1/trnS2.

**Table 2:** Mitochondrial genome sequences of *F. intermediate*.

**Family**	**Taxon**	**Size (bp)**	**Accession number**
Echinostomatidae	Echinostoma caproni 1	14,150	AP017706.1
Echinostomatidae	Hypoderaeum sp.	14,180	KM111525.1
Echinostomatidae	Echinochasmus japonicus	15,865	KP844722.1
Echinostomatidae	Echinostoma hortense	15,048	KR062182.2
Echinostomatidae	Echinostoma paraensei	20,298	KT008005.1
Echinostomatidae	Acanthoparyphium sp.	14,191	MG792058.1
Echinostomatidae	Artyfechinostomum sufrartyfex	14,567	NC_037150.1
Fasciolidae	Fasciola hepatica 1	14,462	AF216697.1
Fasciolidae	Fasciola hepatica 2	14,374	AP017707.1
Fasciolidae	Fasciola gigantica	14,478	KF543342.1
Fasciolidae	Fasciola sp.	14,453	KF543343.1
Fasciolidae	Fascioloides magna	14,047	KU060148.1
Fasciolidae	Fasciolopsis buski	14,833	KX169163.1
Fasciolidae	Fasciola jacksoni	14,952	KX787886.1
Gastrodiscidae	Homalogaster paloniae 1	14,490	KT266674.1
Gastrodiscidae	Homalogaster paloniae 2	15,987	KX169165.1
Gastrothylacidae	Fischoederius elongatus	14,120	KM397348.1
Gastrothylacidae	Gastrothylax crumenifer	14,801	KM400624.1
Gastrothylacidae	Fischoederius cobboldi	14,256	KX169164.1
Heterophyidae	Metagonimus yokogawai	15,258	KC330755.1
Heterophyidae	Haplorchis taichui	15,130	KF214770.1
Notocotylidae	Ogmocotyle sikae	14,307	KR006934.1
Notocotylidae	Ogmocotyle sp.	14,001	KR006935.1
Opisthorchiidae	Opisthorchis felineus	14,277	EU921260.2
Opisthorchiidae	Metorchis orientalis	13,834	KT239342.1
Paragonimidae	Paragonimus westermani 1	14,965	AF219379.2
Paragonimidae	Paragonimus westermani 2	14,244	AF540958.1
Paragonimidae	Paragonimus westermani complex 1 sp.	14,103	KM280646.1
Paragonimidae	Paragonimus ohirai strain Kino	14,818	KX765277.1
Paragonimidae	Paragonimus westermani 3	15,005	KX943544.1
Paramphistomatidae	Paramphistomum cervi 1	14,014	KF475773.1
Paramphistomatidae	Orthocoelium streptocoelium	13,800	KM659177.1
Paramphistomatidae	Calicophoron microbothrioides	14,028	KR337555.1
Paramphistomatidae	Paramphistomum cervi 2	14,023	KT198987.1
Paramphistomatidae	Explanatum explanatum	13,968	KT198989.1
Schistosomatidae	Schistosoma mekongi	14,072	AF217449.1

**Table 3:** Composition and skewness of *F. intermediate* mitogenome.

**Region**	**A%**	**C%**	**G%**	**T%**	**A+T%**	**G+C%**	**AT skew**	**GC skew**
Whole genome	15.17	9.31	27.51	48.02	63.19	36.82	−0.52	0.494
atp6	12.36	10.38	26.71	50.55	62.91	37.09	−0.607	0.44
cob	14.58	8.81	27.08	49.53	64.11	35.89	−0.545	0.509
cox1	14.09	10.31	25.57	50.03	64.12	35.88	−0.561	0.425
cox2	20.75	10.03	27.38	41.84	62.59	37.41	−0.337	0.464
cox3	14.43	9.2	23.13	53.23	67.66	32.34	−0.574	0.431
nad1	13.33	7.55	30.44	48.68	62.01	37.99	−0.57	0.603
nad2	12.43	9.86	25.05	52.66	65.09	34.91	−0.618	0.435
nad3	12.36	8.05	26.44	53.16	65.52	34.48	−0.623	0.533
nad4	13.53	10.01	27.05	49.41	62.94	37.06	−0.57	0.46
nad41	13.64	4.92	29.17	52.27	65.91	34.09	−0.586	0.711
nad5-0	11.84	9.32	26.32	52.52	64.36	35.64	−0.632	0.477
nad6	11.42	9.79	26.34	52.45	63.87	36.13	−0.642	0.458
rrnL	23.91	12.52	28.27	35.29	59.2	40.8	−0.192	0.386
rrnS	21.91	12.37	26.75	38.98	60.89	39.11	−0.28	0.368
trnA	16.92	13.85	30.77	38.46	55.38	44.62	−0.389	0.379
trnC	20	17.14	27.14	35.71	55.71	44.29	−0.282	0.226
trnD	16.92	10.77	29.23	43.08	60	40	−0.436	0.462
trnE	19.12	10.29	23.53	47.06	66.18	33.82	−0.422	0.391
trnF	27.27	12.12	27.27	33.33	60.61	39.39	−0.1	0.385
trnG	18.75	14.06	23.44	43.75	62.5	37.5	−0.4	0.25
trnH	18.75	10.94	34.38	35.94	54.69	45.31	−0.314	0.517
trnI	18.75	14.06	32.81	34.38	53.12	46.88	−0.294	0.4
trnK	20.9	11.94	25.37	41.79	62.69	37.31	−0.333	0.36
trnL1	15.38	13.85	29.23	41.54	56.92	43.08	−0.459	0.357
trnL2	20.31	14.06	29.69	35.94	56.25	43.75	−0.278	0.357
trnM	28.79	13.64	19.7	37.88	66.67	33.33	−0.136	0.182
trnN	22.86	14.29	24.29	38.57	61.43	38.57	−0.256	0.259
trnP	22.06	8.82	30.88	38.24	60.29	39.71	−0.268	0.556
trnQ	18.18	7.58	31.82	42.42	60.61	39.39	−0.4	0.615
trnR	15.52	15.52	22.41	46.55	62.07	37.93	−0.5	0.182
trnS1	12.5	16.07	28.57	42.86	55.36	44.64	−0.548	0.28
trnS2	15.87	14.29	22.22	47.62	63.49	36.51	−0.5	0.217
trnT	20.59	10.29	27.94	41.18	61.76	38.24	−0.333	0.462
trnV	23.44	12.5	21.88	42.19	65.62	34.38	−0.286	0.273
trnW	20.63	11.11	28.57	39.68	60.32	39.68	−0.316	0.44
trnY	16.42	11.94	37.31	34.33	50.75	49.25	−0.353	0.515

### PCGs and Codon Usage

The mitogenome of *F. intermediate* had 12 typical PCGs, containing seven NADP genes (nad1-6 and nad4L), one ATP gene (atp6) and four cytochrome genes (cox1-3 and cob) (Figure 1). The region of PCGs was 8,160 bp in size. The PCGs started with ATG (cox1-3, nad4L), ATA (cob, nad3, nad6), TAG (nad4), ATC (atp6), ATT (nad1-2, nad5), and nine PCGs, terminated by TAA (cox1-3, atp6, nad2, nad4, nad5, nad6 and nad4L). The nad1 and nad3 used TAG as a stop codon whereas the cob terminated by a single T (Table 2).

**Figure 2:**
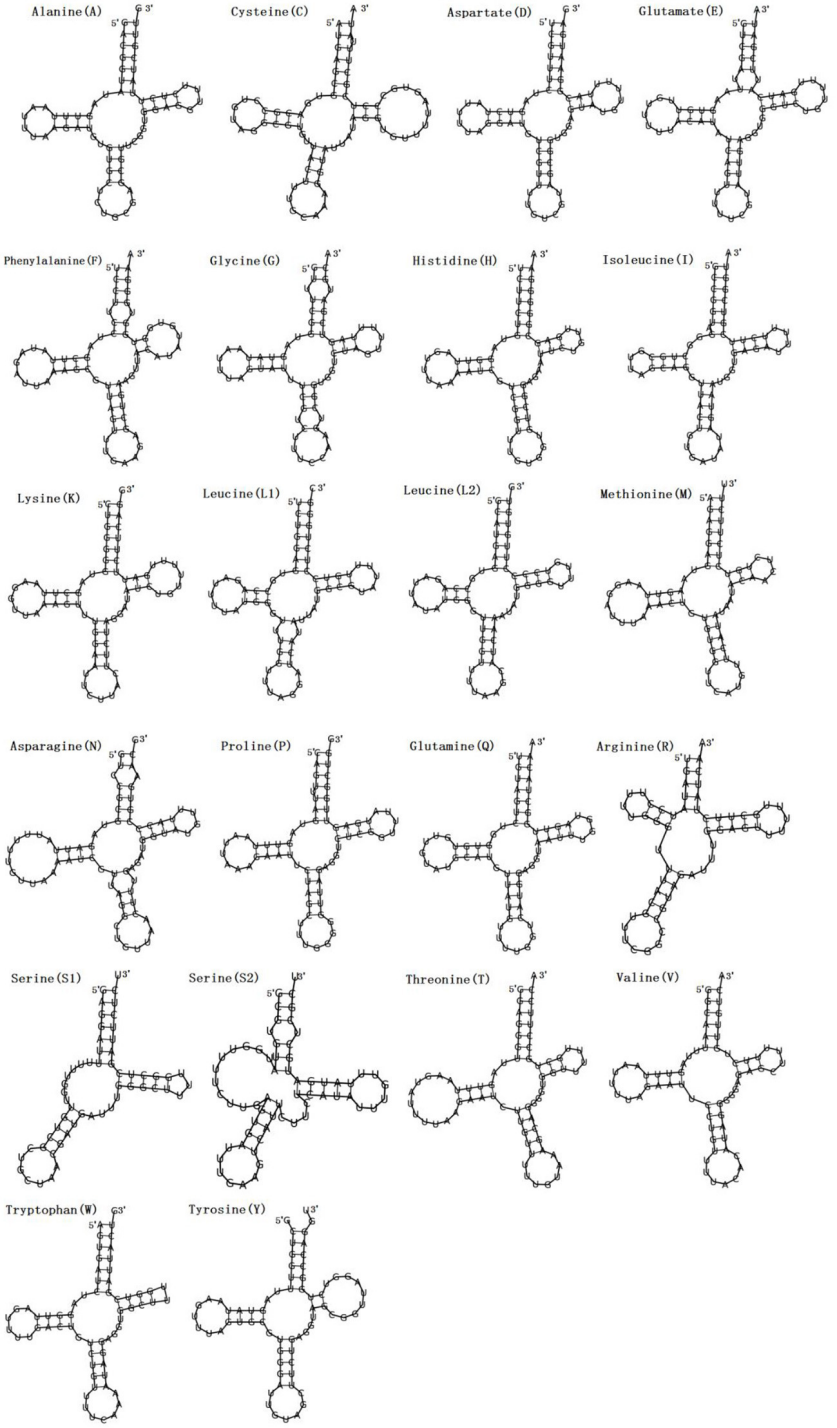
Secondary structures of the 22 transfer RNA genes of *F. intermediate* tRNAs (labeled with the abbreviations of their corresponding amino acids).

The pattern of codon usage and relative synonymous codon usage (RSCU) in the *F. intermediate* mt DNA was studied. A total of 2,716 amino acids were encoded by the *F. intermediate* mitogenome, including the termination codes. The most frequently used amino acids were Leu (16.72%), followed by Val (12.74%), Phe (10.90%), Ser (10.09%) and Gly (8.39%) (Table 4 and Figure 2).

**Table 4:** The codon number and relative synonymous codon usage in *F. intermediate* mitochondrial protein coding genes.

**Amino acid**	**Codon**	**Count**	**RSCU**	**Amino acid**	**Codon**	**Count**	**RSCU**	**Amino acid**	**Codon**	**Count**	**RSCU**	**Amino acid**	**Codon**	**Count**	**RSCU**
Phe(F)	UUU	284	1.92	Ser(S)	UCU	128	3.24	Tyr(Y)	UAU	118	1.90	Cys(C)	UGU	89	1.93
	UUC	12	0.08		UCC	5	0.13		UAC	6	0.10		UGC	3	0.07
Leu(L)	UUA	72	0.38		UCA	5	0.13	End (*)	UAA	1	2	Trp(W)	UGA	14	0.31
	UUG	308	1.62		UCG	20	0.51		UAG	0	0		UGG	77	1.69
Leu(L)	CUU	62	3.35	Pro(P)	CCU	57	2.68	His(H)	CAU	43	1.91	Arg(R)	CGU	38	2.87
	CUC	3	0.16		CCC	3	0.14		CAC	2	0.09		CGC	0	0
	CUA	1	0.05		CCA	5	0.24	Gln(Q)	CAA	6	0.55		CGA	5	0.38
	CUG	8	0.43		CCG	20	0.94		CAG	16	1.45		CGG	10	0.74
Ile(I)	AUU	99	1.89	Thr(T)	ACU	53	3.03	Asn(N)	AAU	45	1.88	Ser(S)	AGU	77	2.66
	AUC	6	0.11		ACC	3	0.17		AAC	3	0.12		AGC	2	0.07
Met(M)	AUA	28	0.48		ACA	2	0.11	Lys(K)	AAA	13	0.55		AGA	7	0.24
	AUG	88	1.52		ACG	12	0.69		AAG	34	1.45		AGG	30	1.03
Val(V)	GUU	246	2.84	Ala(A)	GCU	81	3.38	Asp(D)	GAU	59	1.97	Gly(G)	GGU	147	2.58
	GUC	11	0.13		GCC	5	0.21		GAC	1	0.03		GGC	5	0.09
	GUA	24	0.28		GCA	4	0.17	Glu(E)	GAA	10	0.32		GGA	18	0.32
	GUG	65	0.75		GCG	6	0.25		GAG	53	1.68		GGG	58	1.02

### A+T Skewness and Transfer RNAs

**Figure 3:**
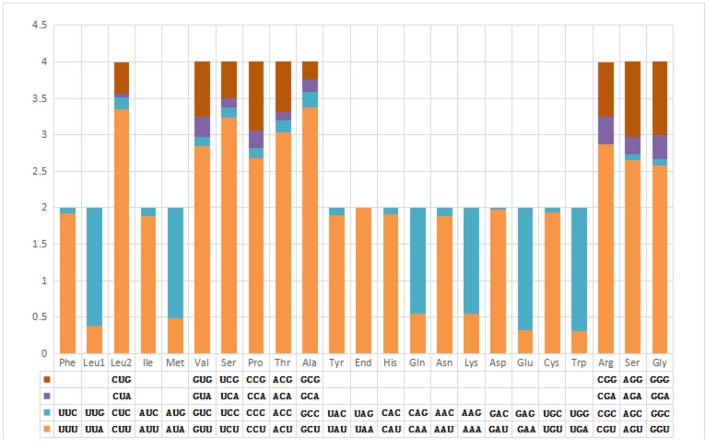
Relative synonymous codon usage in *F. intermediate* mitogenome.

In the mitogenome of *F. intermediate*, the skew of AT was negative and the skew of GC was positive, indicating an obvious bias toward the use of T and A (Table 3). Like most mt DNA, the *F. intermediate* mitogenome contained a set of 22 tRNAs genes. The tRNAs ranged in size from 57 to 71 bp. All the tRNA genes were present on the N strand and all the tRNA genes had the typical cloverleaf structure (Figure 3).

### Phylogenetic Analysis

**Figure 4:**
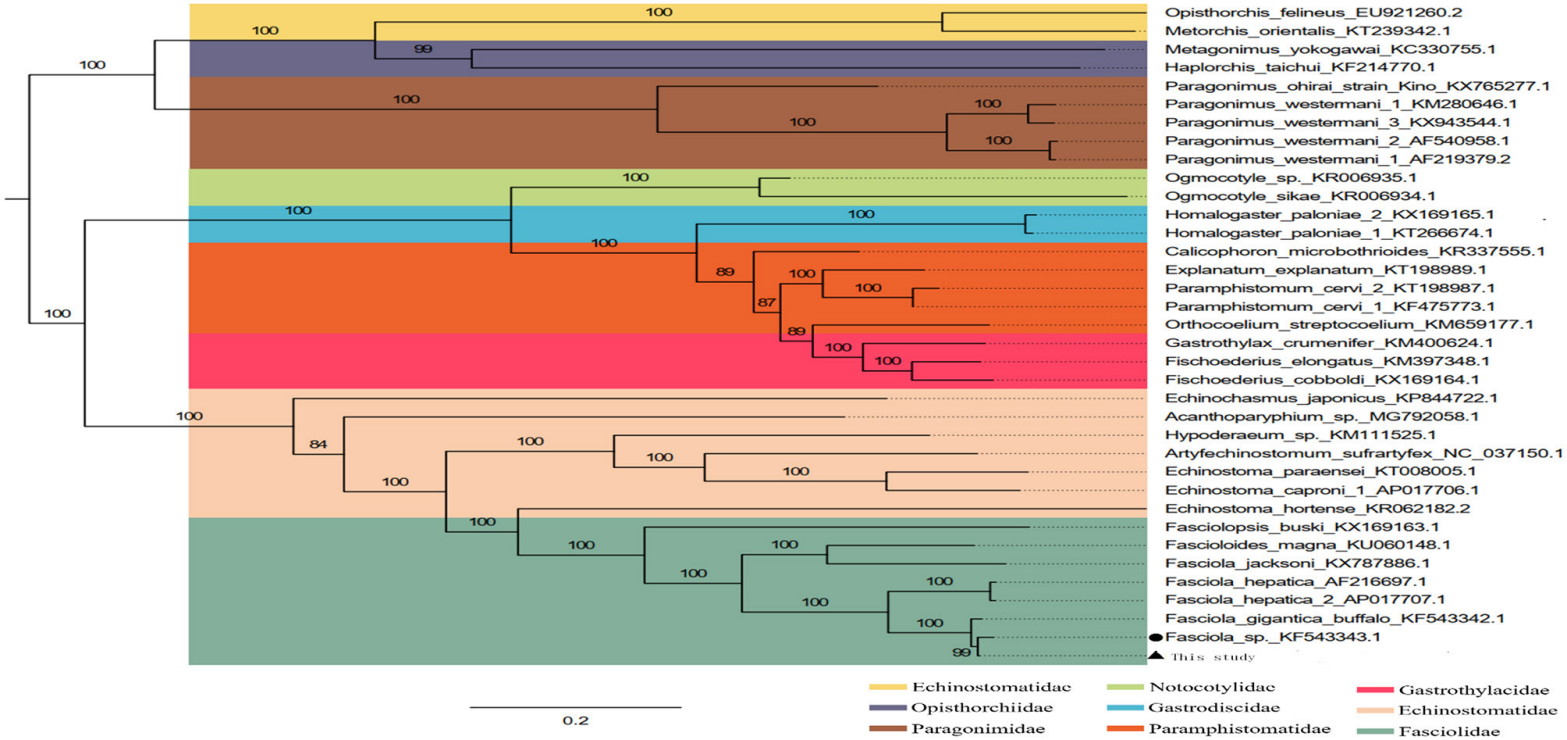
Inferred phylogenetic relationship among the *F. intermediate*. The phylogenetic tree was inferred from the nucleotide sequences of mitogenome by using ML methods (Numbers on branches indicate ML).

The phylogenetic relationship was analyzed based on the concatenated nucleotide sequences of 12 PCGs from 36 Trematoda and one outgroup. The result of analyses, generated a consistent tree topologies (Figure 4).

In this study, the ML analyses showed that each superfamily in the tree formed a monophyletic clade. Obviously, *F. hepatica, F. intermediate*, and *F. gigantica* clustered in one branch in the phylogenetic tree with high nodal support values (Figure 4), indicating that *F. hepatica, F. intermediate*, and *F. gigantica* have a sister group relationship. Additionally, the phylogenetic analyses revealed that *F. hepatica, F. intermediate*, and *F. gigantica* were grouped into one clade in fascioliases within Trematoda, which was consistent with a previous study (4).

### Comparative mt Genomic Analyses With *F. hepatica, F. intermediate*, and *F. gigantica*

The complete mitogenome sequences in this study (*F. intermediate, Fi*, MH621335.1) were 493, 414, and 518 bp shorter than *F. intermediate* from bovine (*fin*, KF543343.1, 14453 bp), *F. hepatica* (*Fh*, AP017707.1, 14374 bp), and *F. gigantica* (*Fg*, KF543342.1, 14478 bp), respectively. A comparison of the nucleotide sequences of each mt gene and the amino acid sequences, conceptually translated from all mt protein-encoding genes of the four flukes, as shown in Tables 5, 6. The sequence difference across the entire mitogenome was 182 nucleotide substitutions between *Fi* and *Fin*, 1, 432 nucleotide substitutions between *Fi* and *Fh*, and 197 nucleotide substitutions between *Fi* and *Fg*. The difference across amino acid sequences of the 12 protein-coding was 42 amino acid substitutions between the *Fi* and *Fin*; 270 amino acid substitutions between *Fi* and *Fh*; and 44 amino acid substitutions between *Fi* and *Fg*, respectively.

**Table 5:** The sequence differences of nucleotide (nt) in each mt gene among *F. intermediate, F. gigantica* and *F. hepatica*.

**Gene/Region**	**Nt sequence length**				**Nt difference**		
	***Fi* (this study)**	***Fin* (KF543343.1)**	***Fh* (AP017707.1)**	***Fg* (KF543342.1)**	***Fi/Fin***	***Fi/Fh***	***Fi/Fg***
atp6	519	519	519	519	29	77	15
nad1	903	903	903	903	27	67	0
nad2	867	867	867	867	31	103	0
nad3	357	357	357	357	20	37	0
nad4	1,269	1,269	1,272	1,269	42	65	20
nad4L	273	273	273	273	0	23	5
nad5	1,566	1,566	1,566	1,566	1	204	28
nad6	453	453	453	453	0	64	18
cox1	1,542	1,542	1,542	1,542	5	143	26
cox2	603	603	603	603	1	65	10
cox3	642	642	642	642	1	83	17
cytb	1,113	1,113	1,113	1,113	2	90	23
rrnL	986	986	987	986	0	99	10
rrnS	769	769	763	771	0	92	10
trna	1,417	1,413	1,426	1,414	23	171	11
at-loop1	176	176	187	174	0	49	4
at-loop2	329	841	732	841	−	−	−

**Table 6:** Amino acid (aa) sequence differences in each mt gene among *F. intermediate, F. gigantica*, and *F. hepatica*.

**Gene/Region**	**Number of aa**				**aa difference**		
	***Fi* (this study)**	***Fin* (KF543343.1)**	***Fh* (AP017707.1)**	***Fg* (KF543342.1)**	***Fi/Fin***	***Fi/Fh***	***Fi/Fg***
atp6	172	172	172	172	9	25	6
nad1	300	300	300	300	9	20	0
nad2	288	288	288	288	5	31	0
nad3	118	118	118	118	1	9	0
nad4	422	422	423	422	10	38	7
nad4L	90	90	90	90	0	4	1
nad5	521	521	521	521	1	58	5
nad6	150	150	150	150	0	9	11
cox1	513	513	513	513	4	24	3
cox2	200	200	200	200	0	10	1
cox3	213	213	213	213	1	25	5
cytb	370	370	370	370	2	17	5

## Discussion

Fasciolosis is a zoonotic disease belonging to water-borne trematodes. The previous studies showed the prevalence of fasciolosis in Africa with the highest range in cattle (1.2–91.0%) and the lowest in sheep (0.19–73.7%). In America the highest range was in goats (24.5–100%) and the lowest in cattle (3.0–66.7%) in America. In Asia the highest range in cattle (0.71–69.2%) and lowest in goat (0.0–47.0%). In Australia, the highest range was in cattle (26.5–81.0%), and the lowest range was in sheep (5.5–52.2%). While in Europe, the highest range was in cattle (0.12–86.0%) and the lowest in goats (0.0–0.8%) (20). Annually 2000 million dollars are lost because of helminthic infection (21).

*F. hepatica* and *F. gigantica* are considered two effective species in the genus Fasciola. However, the researchers found that there was also an “intermediate type” of Fasciola (*F. intermediate*). This “intermediate type” of Fasciola was first discovered in Japan and was subsequently reported in China and South Korea (22). Many studies on Fascioliasis have limited information about the Fasciola types in yaks. Genome-based molecular identification has been more commonly employed to identify biological types in recent years due to advances in biotechnology. mt DNA is an ideal genetic marker due to the simple and stable structure, reflecting the maternal genetic background (23).

Our study showed that *F. intermediate* from yak and *F. intermediate* from bovine belonged to the same branch and it was to be similar to *F. gigantica* compared with the *F. hepatica* through phylogenetic analysis. We compared the whole mitochondrial genome between *Fi, Fin, Fg, and Fh*. The results showed that *Fi* was no base site mutations in rrnL and rrnS compared with *Fin*, 10 base site mutations in rrnL and rrnS compared with *Fg*. Compared with *Fh, Fi* there were 99 base site mutations in rrnL and 92 base site mutations in rrnS, respectively. Hence, it would be the ideal genetic marker to distinguish the three species of Fasciola. Meanwhile, *Fi* had a deletion of nearly 500 bp in the AT-loop area, compared with *Fin, Fg*, and *Fh*, which may be due to the adaptability to plateau for *Fi*.

## Conclusion

In this study, we sequenced the complete 13,960 bp mitogenome of *F. intermediate* from yaks, in which 36 genes (12 PCGs, 22tRNA genes and 2 rRNA genes) were located as a typical of Trematoda mitogenome. All PCGs were initiated by ATN codon, the cob genes had incomplete stop codons consisting of just T, and the other 11 PCGs stop with the canonical TAA or TAG. The AT-skew were negative, and the GC-skew were positive in the mitogenomes of *F. intermediate*, consistent with most sequenced Trematoda. The phylogenetic analyses support that *F. intermediate* from yaks was the same as the *F. intermediate* from bovine. This study would provide a basis for the accurate prevention, diagnosis, and treatment of Fasciolosis in yaks.

## Data Availability Statement

The datasets presented in this study can be found in online repositories. The names of the repository/repositories and accession number(s) can be found in the article/supplementary material.

## Ethics Statement

The samples were collected under the permission of the relevant institutions. All procedures were approved and performed by Laboratory Animals Research Centre of Hubei province and Qinghai province in P.R. China, and the Ethics Committee of Huazhong Agricultural University, China (Permit number: 4200695757). All animal experiments and procedures were conducted under the relevant procedures of Proclamation of the Standing Committee of Hubei People's congress (PSCH No. 5), China.

## Author Contributions

XG, JZ, and JL conceived and designed the study. XG, SZ, ZZ, KL, and XK executed the experiment and analyzed the sera and tissue samples. XG, SZ, CQ, and YL analyzed the data. XG and DW finished the first draft. MK finished the revision. All authors interpreted the data, critically revised the manuscript for important intellectual contents, and approved the final version.

## Funding

This study was supported by the Chinese Agricultural Research Systems (CARS-37) and the major science and technology projects of Tibet Autonomous Region (XZ202101ZD0002N).

## Conflict of Interest

The authors declare that the research was conducted in the absence of any commercial or financial relationships that could be construed as a potential conflict of interest.

## Publisher's Note

All claims expressed in this article are solely those of the authors and do not necessarily represent those of their affiliated organizations, or those of the publisher, the editors and the reviewers. Any product that may be evaluated in this article, or claim that may be made by its manufacturer, is not guaranteed or endorsed by the publisher.
